# Does suberin accumulation in plant roots contribute to waterlogging tolerance?

**DOI:** 10.3389/fpls.2013.00178

**Published:** 2013-06-17

**Authors:** Kohtaro Watanabe, Shunsaku Nishiuchi, Konstantin Kulichikhin, Mikio Nakazono

**Affiliations:** Laboratory of Plant Genetics and Breeding, Graduate School of Bioagricultural Sciences, Nagoya UniversityNagoya, Japan

**Keywords:** apoplastic barrier, exodermis, hypodermis, suberin, suberin composition, radial oxygen loss, waterlogging

## Abstract

Plants that are adapted to waterlogged conditions develop aerenchyma in roots for ventilation. Some wetland plant species also form an apoplastic barrier at the outer cell layers of roots that reduces radial oxygen loss (ROL) from the aerenchyma and prevents toxic compounds from entering the root. The composition of the apoplastic barrier is not well understood. One potential component is suberin, which accumulates at the hypodermal/exodermal cell layers of the roots under waterlogged soil conditions or in response to other environmental stimuli. However, differences in suberin content and composition between plant species make it difficult to evaluate whether suberin has a role in preventing ROL. In this article, we summarize recent advances in understanding apoplastic barrier formation in roots and, between various plant species, compare the chemical compositions of the apoplastic barriers in relation to their permeability to oxygen. Moreover, the relationship between suberin accumulation and the barrier to ROL is discussed.

## Introduction

Waterlogging is defined as a condition of the soil in which excess water limits gas diffusion and inhibits plant growth. Oxygen diffusivity in water is approximately 10,000 times slower than it is in air, and the flux of O_2_ into soils is approximately 320,000 times less when the soil pores are filled with water than when they are filled with gas (Armstrong and Drew, 2002). Moreover, oxygen in waterlogged soil is consumed rapidly by soil microorganisms and plant roots (Drew and Lynch, 1980). As a result, the growth of anaerobic soil microorganisms is accelerated, leading to the appearance of harmful microbial metabolic products such as organic acids (Jackson and Taylor, 1970; Conrad and Klose, 1999). Soil redox potential is also rapidly decreased, resulting in the accumulation of phytotoxic compounds such as sulfides and reduced forms of minerals (e.g., Mn^2+^ and Fe^2+^) (Laanbroek, 1990). Plants that are adapted to waterlogged soil conditions form aerenchyma, which permits ventilation between well-aerated shoots and waterlogged roots. Additionally, some waterlogging-tolerant species develop an apoplastic transport barrier at the outer cell layers in the root. This barrier can be also induced by other environmental stresses, such as elevated salinity and drought (Enstone et al., 2003), to reduce of the transport of water, solutes, and gases from the medium to the root and *vice versa*. When the soil is waterlogged, the barrier can minimize the loss of O_2_ to the surrounding environment, called radial oxygen loss (ROL), thereby enhancing longitudinal diffusion of oxygen toward the root apex (Armstrong, 1979; Colmer, 2003a). The barrier also impedes the penetration of soil-derived toxins, such as reduced metal ions, into the roots (Armstrong and Armstrong, 2005; Greenway et al., 2006). Mathematical modeling suggests that oxygen diffusion to the apex largely depends on the development of aerenchyma in the plant root (Armstrong, 1979; Armstrong and Beckett, 1987). Therefore, in the presence of extensive aerenchyma, the function of the apoplastic barrier may be more important for restricting the entry of phytotoxins than for improving oxygen diffusion to the apex (Armstrong, 1979).

**Figure 1:**
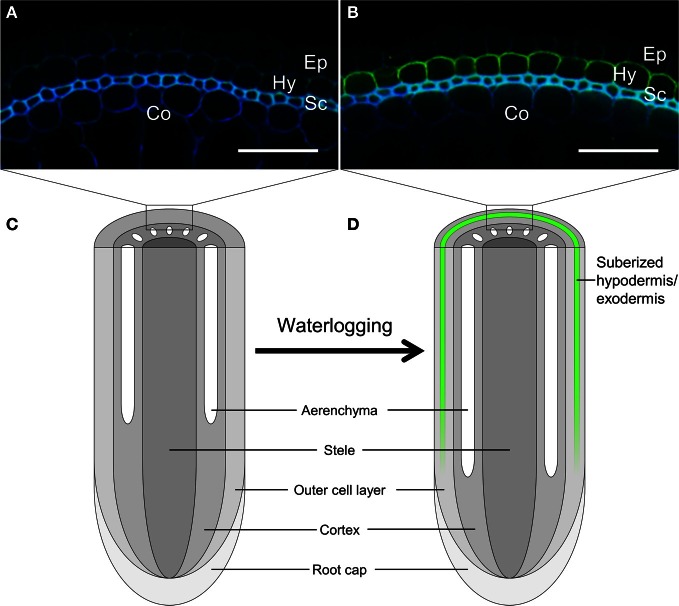
**Induction of suberization at the hypodermis/exodermis of rice root under stagnant deoxygenated conditions.** Nine-day-old rice plants were grown in aerated solution **(A)** or stagnant deoxygenated solution **(B)** for 14 days. Basal parts (60 mm from root tip) of the adventitious roots were sliced into 80-μm-thick sections. The sections were cleared by incubating them in lactic acid saturated with chloral hydrate at 70°C for 1 h. Suberin was stained yellow green, especially in the hypodermis/exodermis, with Fluorol Yellow 088 at room temperature for 1 h. The section also showed autofluorescence in blue. Ep, epidermis; Hy, hypodermis/exodermis; Sc, sclerenchyma; Co, cortex. Scale bars = 50 μm. **(C,D)** Schematic diagram of longitudinal view of rice root under aerobic conditions **(C)** or under stagnant deoxygenated conditions **(D)**. Suberized hypodermis/exodermis is shown by green line at **(D)**.

The apoplastic barrier forms as the result of enhanced accumulation of suberin at hypodermal/exodermal cell walls (Figure 1). Suberin is one of the main barrier biopolymers in plants, and is deposited in cell walls to separate living plant tissue from unfavorable environments or to separate different tissues inside the plant during development (Kolattukudy, 2001; Enstone et al., 2003; Schreiber, 2010). Suberin was found to be deposited in the outer cell layers of roots in stagnant deoxygenated medium in several plant species (De Simone et al., 2003; Soukup et al., 2007; Garthwaite et al., 2008; Kotula et al., 2009a).

In this review, we summarize what is known about the induction and function of the apoplastic barrier in plant roots under waterlogged conditions, and examine the relation between the chemical composition of the barrier and its permeability to O_2_ in several plant species. Finally, we discuss future directions for understanding the composition of the apoplastic barrier and the potential contribution of the apoplastic barrier to improvement of waterlogging tolerance of crops.

## Induction of apoplastic barrier in plant roots

Accumulation of suberin is manifested by the presence of a Casparian strip and the development of suberin lamellae in the hypodermis/exodermis or the endodermis. The maturation processes of the Casparian strip and suberin lamellae in the hypodermis/exodermis differ between plant species and between growth conditions (Enstone et al., 2003). Suberization of the outer cell layers in rice (*Oryza sativa*) root occurs under stagnant deoxygenated conditions (which mimic low-oxygen conditions in waterlogged soils) (Figure 1; Wiengweera et al., 1997; Kotula et al., 2009a) or after exposure to short-chain carboxylic acids (Armstrong and Armstrong, 2001) or sulfide (Armstrong and Armstrong, 2005). Short-chain carboxylic acids (Conrad and Klose, 1999) and sulfide (Jacq et al., 1991) are known to accumulate around plant roots by reduction reactions and by the activity of anaerobic bacteria under waterlogged soil conditions. Some short-chain carboxylic acids are phytotoxins that damage cell membranes and thus may induce the suberization of plant roots (Armstrong and Armstrong, 2001).

In the absence of suberin, the plant cell wall is porous with pore sizes of 3.5–5.2 nm (Carpita et al., 1979) that allow water, salts, and gases to move freely between the plant and surrounding environment. Increased amounts of suberin and lignin are thought to make strong barriers by reducing the diameter of pores in the apoplastic barriers (Hose et al., 2001). The Casparian strip and suberin lamella restrict apoplastic transfer of Ca^2+^ (hydrated ionic radius: approximately 412 pm; Volkov et al., 1997) and Mg^2+^ (hydrated ionic radius: approximately 428 pm; Volkov et al., 1997) in barley (*Hordeum vulgare*) (Ferguson and Clarkson, 1976) and maize (*Zea mays*) (Maas and Ogata, 1971; Ferguson and Clarkson, 1975). Formation of an apoplastic barrier in the hypodermal/exodermal cells in roots of rice grown in stagnant deoxygenated solution for 17–27 days decreased the permeability of the outer part of the root to water and NaCl (Ranathunge et al., 2011). The decrease of permeability was greater for NaCl than for water, which suggests that the wall pore size in suberized cell walls is large enough to pass water (radius of an H_2_O molecule: approximately 137 pm; Zhang and Xu, 1995) but not Na^+^ solvated by water molecules (hydrated ionic radius: approximately 358 pm; Volkov et al., 1997). The strengthened apoplastic barriers also appear to prevent the entry of reduced phytotoxic compounds [e.g., Fe^2+^ (approximately hydrated ionic radius 300 pm; Kielland, 1937) and H_2_S (approximately radius 193 pm; Kammeyer and Whitman, 1972)] and diffusion of gases [e.g., O_2_ (approximately radius 140 pm; Kammeyer and Whitman, 1972)] (Armstrong et al., 2000). However, it should be noted that the size and number of pores in suberized cell walls could be reduced with increasing level of suberization, and that other factors, such as the electrical charge of the cell wall, can limit the flow of substances. The permeability of the apoplastic barrier depends on growth conditions and the length of time in waterlogged soil.

Although suberin accumulation reduces the permeability of the apoplastic barrier to most substances, the tight apoplastic barrier to ROL in root basal zones does not reduce NO^−^_3_ uptake from aerobic solutions in rice grown in stagnant nutrient solution (Rubinigg et al., 2002). This may be because plasmodesmatal connections, as observed in electron micrographs, are still operational in suberized tissue of the exodermis of adventitious roots from rice grown in hydroponic culture (Clark and Harris, 1981; Rubinigg et al., 2002).

## Some aspects of the chemical composition of apoplastic barrier in relation to its ability to prevent ROL

Schreiber et al. (1999) developed a protocol for the analysis of the apoplastic barrier in plant roots consisting of enzymatic digestion of cortical tissue, mechanical isolation of the remaining hypodermal/exodermal tissue, solvent extraction, chemical degradation of lignin and suberin, and analysis of the released monomers by gas chromatography-mass spectrometry (GC-MS). The apoplastic barriers of several plant species have been analyzed with this protocol (De Simone et al., 2003; Soukup et al., 2007; Kotula et al., 2009a). The results of those studies, which also include ROL measurements, are summarized in Table 1. In the following, we briefly discuss some aspects of the chemical composition of the barrier in relation to its ability to prevent ROL.

**Table 1:**
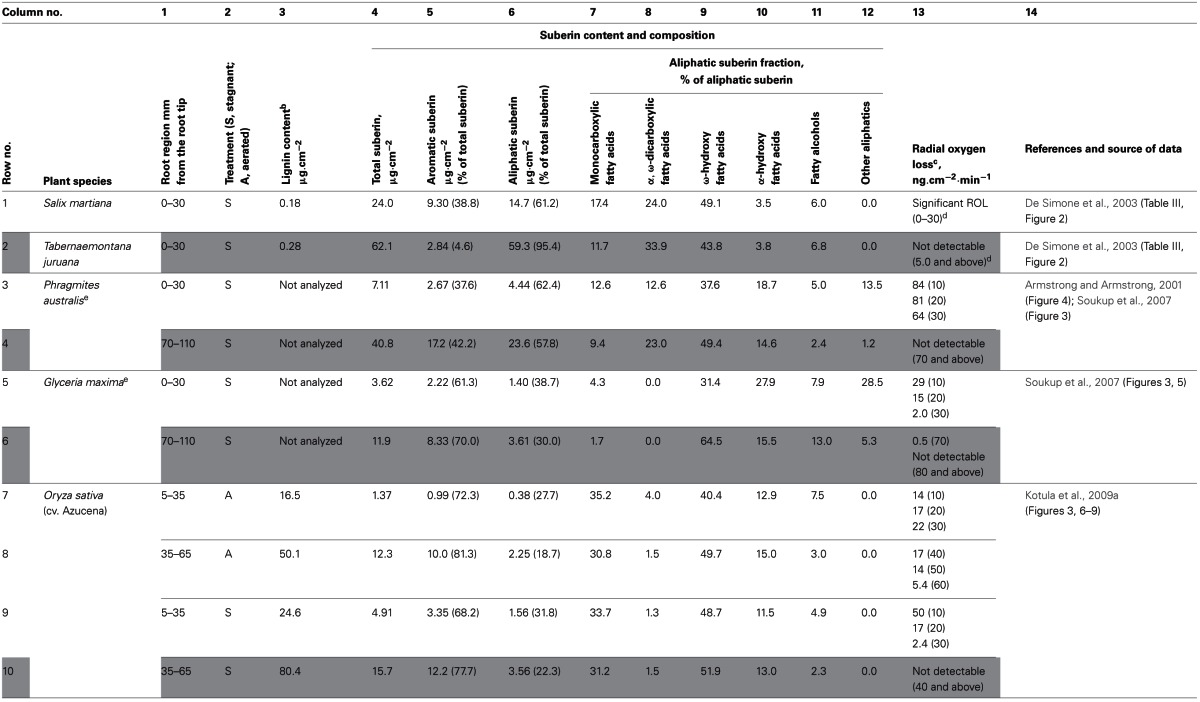
**Chemical composition of apoplastic barrier to Radial Oxygen Loss in various plant species^a^**.

^a^Cases in which a strong barrier to ROL is formed are marked in gray and cases that have significant ROL across the root surface are not marked.

^b^The amount of lignin and suberin has been recalculated and expressed in μg·cm^−2^ of root surface where necessary.

^c^Radial Oxygen Loss (except for Tabernaemontana juruana and Salix martiana) is expressed in ng·cm^−2^.min^−1^. The number in parentheses is the distance (in mm) from the root tip to the point where ROL was measured.

^d^Root cortex oxygen concentration and root surface concentration rather than ROL were measured for Tabernaemontana juruana and Salix martiana (De Simone et al., 2003, Figures 5, 6). Root surface oxygen concentration for T. juruana was above zero just at the region 0–5 mm from the root tip, reaching a maximum of 0.4 mg.l^−1^ at 2 mm from the apex. The surface O_2_ concentration in the region above 5 mm from the root tip was below detection limit. Nevertheless, the internal oxygen concentration measured at 10–15 mm from the apex was 2.0 mg·l^−1^. This means that the barrier to ROL was very tight. In the case of S. martiana, the root surface concentration of oxygen was close to its concentration in the root cortex, which shows that if an apoplastic barrier was present, it would not be strong enough to prevent oxygen release from the root to anaerobic environment.

^e^For Phragmites australis and Glyceria maxima the results are for stagnant treatment only. For both species, the aliphatic suberin quantity and composition in plants growing in stagnant conditions were not significantly different from those in plants growing well aerated conditions. The profile of ROL for roots of P. australis plants growing in aerated conditions was similar to that of plants growing in stagnant conditions indicating that P. australis has a constitutive barrier to ROL. On the other hand, roots of G. maxima plants growing in aerated conditions did not form a tight barrier to ROL.

### Total suberin content

In several plant species [*Phragmites australis* (Table 1, rows 3–4), *Glyceria maxima* (Table 1, rows 5–6), and rice (Table 1 rows 9–10)], the suberin contents in the peripheral regions at several points along the root (Table 1, column 4) were inversely proportional to the ROL at those points (Table 1, column 13). On the other hand, if only the data obtained when ROL was not detectable are considered (gray rows in Table 1), the species differ dramatically in the amount of suberin. For example, the peripheral region of *Tabernaemontana juruana* root (Table 1, row 2, column 4) contained 5.5 times more suberin than that of *G. maxima* (Table 1, row 6, column 4), 4 times more suberin than the outer part of rice root (Table 1, row 10, column 4), and 1.5 times more suberin than in those tissues of *P. australis* (Table 1, row 4, column 4), whereas ROL was not measurable in all cases. This variation may be due to differences in spatial distribution of suberin among cell layers, as well as in chemical composition of suberin deposits in different species (Schreiber et al., 2005a).

### Suberin composition and ability of barrier to prevent ROL

Among the entries in Table 1, where ROL is below the detection limit (rows shaded gray), those with a higher percentage of aromatic suberin (Table 1, column 5) tend to have a lower total suberin content (Table 1, column 4). Pearson's correlation coefficient for these two variables calculated from the data in Table 1 (total suberin content 62.1, 40.8, 11.9, 15.7 μg.cm^−2^ and percentage of aromatic suberin 4.6, 42.2, 70.0, 77.7% for *T. juruana, P. australis, G. maxima*, and *O. sativa*, respectively) is −0.982. The absolute value of the coefficient is higher than the critical value for *P* = 0.05 and *n* = 4 (0.950), indicating that total suberin content and percentage of aromatic suberin are negatively correlated.

### Composition of aliphatic domain of suberin

The permeability of suberin to water and solutes was found to be mostly determined by the aliphatic domain of suberin (Hose et al., 2001). In *P. australis* and *G. maxima* (Soukup et al., 2007), as well as in rice (Kotula et al., 2009a), the composition of aliphatic suberin isolated from the peripheral part of the root after incubation in stagnant deoxygenated medium was similar to that of suberin isolated from the peripheral part of the root of well-aerated plants. Most aliphatic monomers that are released after suberin decomposition belonged to one of the following five classes: monocarboxylic fatty acids, α,ω-dicarboxylic fatty acids, ω-hydroxy fatty acids, α-hydroxy fatty acids, and fatty alcohols (Table 1, column 7–11). Similar sets of monomers were found in suberin isolated from various herbaceous plant sources, such as wound periderm of potato (Schreiber et al., 2005b; Yang and Bernards, 2006), *Arabidopsis* root tissues (Franke et al., 2005), and *Arabidopsis* and *Brassica napus* seeds (Molina et al., 2006). Among monomer classes, ω-hydroxy fatty acids (Table 1, column 9) were the most abundant class of suberin monomers for all plant species presented in Table 1, accounting for up to 64.5% of all suberin monomers. The abundances of other classes differ substantially among plant species, which makes it difficult to determine whether the composition of the aliphatic domain of suberin affects the permeability of the barrier to O_2_.

### Does lignin contribute to the ROL barrier?

The tight apoplastic barrier in *T. juruana* is almost exclusively built of suberin. On the other hand, in rice, the outer cell layers have also a significant amount of lignin (5 times higher than those of suberin; Table 1, row 10, columns 3–4), although it is unclear whether the lignin contributes to the ROL barrier. In the outer part of the rice root, lignin is predominantly concentrated in lignified sclerenchyma. Lignin content was measured (Kotula et al., 2009a), but whether lignified sclerenchyma can act as the ROL barrier has not been evaluated experimentally. In roots of *P. australis*, sclerenchyma is represented by several layers of cells called a sclerenchymatous ring. Histochemical staining revealed that the walls of these cells were strongly lignified. Nevertheless, the sclerenchymatous ring did not provide a significant barrier to diffusion of periodic acid and is also unlikely to be a barrier to diffusion of smaller molecules such as O_2_ (Soukup et al., 2007).

## Relationship between suberin accumulation and resistance to ROL

Suberin accumulation has been suggested to increase the resistance to O_2_ leakage. However, increased resistance to ROL is not always accompanied by an increase of suberin deposition in the plant root. Shiono et al. (2011) reported that when aerobically grown rice plants were treated under stagnant deoxygenated solution for 2 days, a tight ROL barrier was formed in long adventitious roots, whereas suberin and lignin deposits were undetectable by histochemical staining. Subsequently, suberin deposits increased prior to changes in lignin deposits. On the other hand, high-density granules were observed at intercellular spaces between the hypodermal/exodermal cells and also between the sclerenchymatous cells in long roots treated under stagnant deoxygenated conditions for 2 days, whereas such granules were absent in roots growing under aerated conditions. Shiono et al. (2011) speculated that microstructural packing of intercellular spaces at the hypodermis/exodermis may be involved in ROL barrier formation and that suberin deposition may be more important than lignin deposition.

In waterlogging-tolerant *Zea nicaraguensis*, stagnant deoxygenated growth conditions induced early development of hypodermal/exodermal suberin lamellae as well as deposition of lignin at the epidermis in adventitious roots (Abiko et al., 2012). On the other hand, in waterlogging-intolerant maize, many of the hypodermal/exodermal cells developed suberin lamellae, but an increase in lignin was not detected (Abiko et al., 2012). The absence of an ROL barrier in spite of suberin deposition in maize roots may be due to the presence of non-suberized passage cells in the hypodermal/exodermal cell layer (Armstrong et al., 2000) and/or the special arrangement of suberin deposition within cell walls (Schreiber et al., 2005a; Ranathunge et al., 2011).

Roots of *G. maxima* grown under stagnant deoxygenated conditions develop an ROL barrier (Soukup et al., 2007). However, the formation of the ROL barrier was not accompanied by an increase of suberin content in roots or by changes in suberin composition, which suggests that the net accumulation of suberin components in the hypodermal/exodermal layers do not necessarily reflect the barrier properties of impregnated cell walls (Schreiber et al., 2005a; Soukup et al., 2007).

Histochemical staining is less sensitive than quantitative chemical analysis (De Simone et al., 2003), and may depend on the composition, molecular and spatial arrangements, and differences in molecular context in suberin lamellae (Soukup et al., 2007). On the other hand, current suberin quantitative and qualitative data (Table 1) are insufficient to tell whether the chemical composition of the apoplastic barrier affects ROL. The contributions of lignification and cell wall proteins to the apoplastic barrier remain unclear (Schreiber et al., 2005a). Moreover, the arrangements as well as the contents of suberin monomers may play a role in the formation of the ROL barrier under waterlogged conditions in plants (Ranathunge et al., 2011). Thus, further analyses of the spatial arrangement of suberin deposition within the cell wall are required to elucidate the components of the ROL barrier.

Oxygen consumption by respiration in the outer part of the root also significantly affects ROL (Armstrong et al., 2000; Garthwaite et al., 2008; Kotula et al., 2009b). To determine the real effect of the barrier on ROL, the respiration rate or the number of respiring cell layers in the outer part of the root should be taken into account (Garthwaite et al., 2008; Kotula and Steudle, 2009). For example, when cell respiration was stopped by HCl treatment, the oxygen permeability coefficient of rice root segments was about twice that of non-treated roots (Kotula et al., 2009b), and in the case of *Hordeum marinum*, reducing cell respiration by cooling to 4°C revealed that the cell respiration reduces ROL and improves the apparent tightness of the barrier at 20°C (Garthwaite et al., 2008).

## Challenges and perspectives

Although the ROL barrier was first reported almost 50 years ago and was presumed to be due to suberin deposition (Armstrong, 1964, 1967), the chemical nature and induction of the barrier have been studied in detail only in the last 15 years. Suberin deposition in the outer cell layers of roots is presently suspected to take an important part in the formation of the apoplastic barrier to phytotoxins and gases, and thus it can contribute to waterlogging tolerance. However, differences in spatial patterns of suberin deposition and differences in suberin content and composition between plant species make it difficult to determine to what extent suberin deposition contributes to the barrier formation and how the chemical composition of suberin affects barrier properties. In addition, much remains to be learned about the molecular mechanism of suberin accumulation. One approach to answering these questions is to use model plants for which mutants and transformants that differ in suberin contents and compositions can be easily produced. Rice may be useful for this purpose because many molecular and genetic tools have been developed and it forms a tight barrier in roots under stagnant deoxygenated conditions (Colmer, 2003b; Kotula et al., 2009a; Shiono et al., 2011). Thus, genes related to suberin biosynthesis and ROL barrier formation could be identified and analyzed using rice. Another approach is to focus on the strengthening of the apoplastic barrier that can be induced by chemical stimulants, such as sulfides and short-chain organic acids, in the surrounding environment (Armstrong and Armstrong, 2001, 2005). This would allow suberin content and composition to be varied by chemical stimulants, which in turn would make it possible to determine the effects of suberin content and composition on the tightness of apoplastic barrier to ROL.

Many commercially important crops, such as wheat (*Triticum aestivum*) and maize, are not able to form a tight ROL barrier, which can result in a reduction of yield if the soil becomes waterlogged. On the other hand, wild plants, *H. marinum* and *Z. nicaraguensis*, which can be crossed with wheat and maize, respectively, accumulate suberin in response to waterlogged conditions and form a tight ROL barrier (Malik et al., 2011; Abiko et al., 2012). Some amphiploids between wheat (cv. Chinese Spring) and *H. marinum* were able to form an ROL barrier and were more tolerant to waterlogging than wheat (Malik et al., 2011). In addition, genetic analyses using a cross between *Z. nicaraguensis* and maize (inbred line Mi29) have revealed potential quantitative trait loci (QTL) related to waterlogging tolerance (Mano and Omori, 2008, 2009; Mano et al., 2009, 2012). Such genetic analyses provide a promising approach to understanding the mechanism of ROL barrier formation and to improving the waterlogging tolerance of crops.

### Conflict of interest statement

The authors declare that the research was conducted in the absence of any commercial or financial relationships that could be construed as a potential conflict of interest.
